# Author Correction: Adhesive anti-fibrotic interfaces on diverse organs

**DOI:** 10.1038/s41586-025-09311-5

**Published:** 2025-07-11

**Authors:** Jingjing Wu, Jue Deng, Georgios Theocharidis, Tiffany L. Sarrafian, Leigh G. Griffiths, Roderick T. Bronson, Aristidis Veves, Jianzhu Chen, Hyunwoo Yuk, Xuanhe Zhao

**Affiliations:** 1 Department of Mechanical Engineering, Massachusetts Institute of Technology, Cambridge, MA USA; 2 Joslin-Beth Israel Deaconess Foot Center and The Rongxiang Xu, MD, Center for Regenerative Therapeutics, Beth Israel Deaconess Medical Center, Harvard Medical School, Boston, MA USA; 3 Department of Thoracic Surgery, Mayo Clinic, Rochester, MN USA; 4 Department of Cardiovascular Medicine, Mayo Clinic, Rochester, MN USA; 5 Department of Immunology, Harvard Medical School, Boston, MA USA; 6 Koch Institute for Integrative Cancer Research and Department of Biology, Massachusetts Institute of Technology, Cambridge, MA USA; 7 Department of Civil and Environmental Engineering, Massachusetts Institute of Technology, Cambridge, MA USA; 8 Present Address: SanaHeal, Cambridge, MA USA

**Keywords:** Biomedical engineering, Implants

Correction to: *Nature* https://doi.org/ 10.1038/s41586-024-07426-9 Published online 22 May 2024

In the version of the article initially published, in Fig. 3d, the immunofluorescence staining image for vimentin on day 7 post-implantation was inadvertently sourced from the immunofluorescence staining image for vimentin on day 14 post-implantation. The image has now been corrected in the HTML and PDF versions of the article, as seen in Fig. 1, below. The change does not alter the results or conclusions of the paper.

**Fig. 1:**
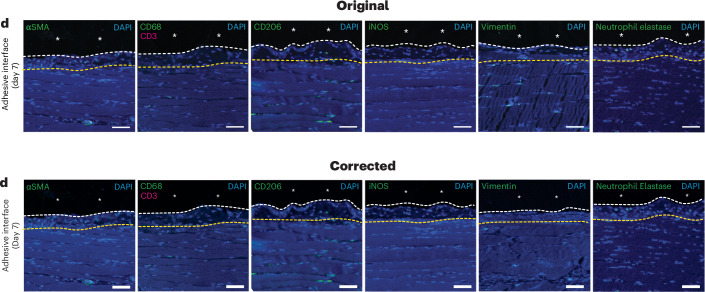
Original and corrected Fig. 3d .

