# Supplementary material for: Adhesive anti-fibrotic interfaces on diverse organs
Source: Nature. 2024 May 22;630(8016):360–7. doi: 10.1038/s41586-024-07426-9 (PMC11168934; doi:10.1038/s41586-024-07426-9)
Supplement: Supplementary file 1 — Supplementary Figs. 1–6 and Tables 1 and 2. [file 41586_2024_7426_MOESM1_ESM.pdf]

nature portfolio
https://doi.org/10.1038/s41586-024-07426-9

Supplementary information

Adhesive anti-fibrotic interfaces on diverse organs

In the format provided by the authors and unedited

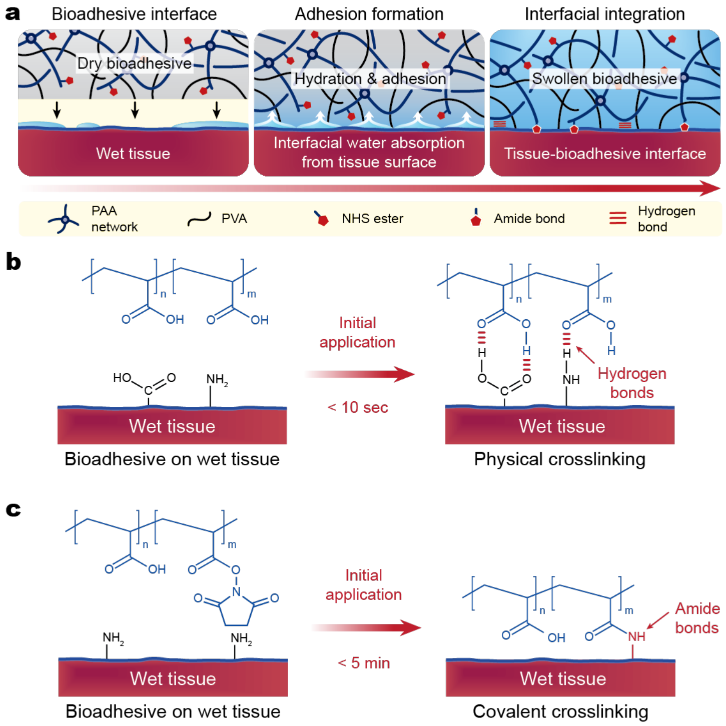

Supplementary Fig. 1 I Adhesion mechanism and chemistry of the adhesive implant. a, Schematic illustrations for adhesion of the adhesive implant to wet tissues. **b**, Schematic illustrations for physical crosslinking between the adhesive implant and the tissue by hydrogen bonds. **c**, Schematic illustrations for covalent crosslinking between the adhesive implant and the tissue by amide bonds.

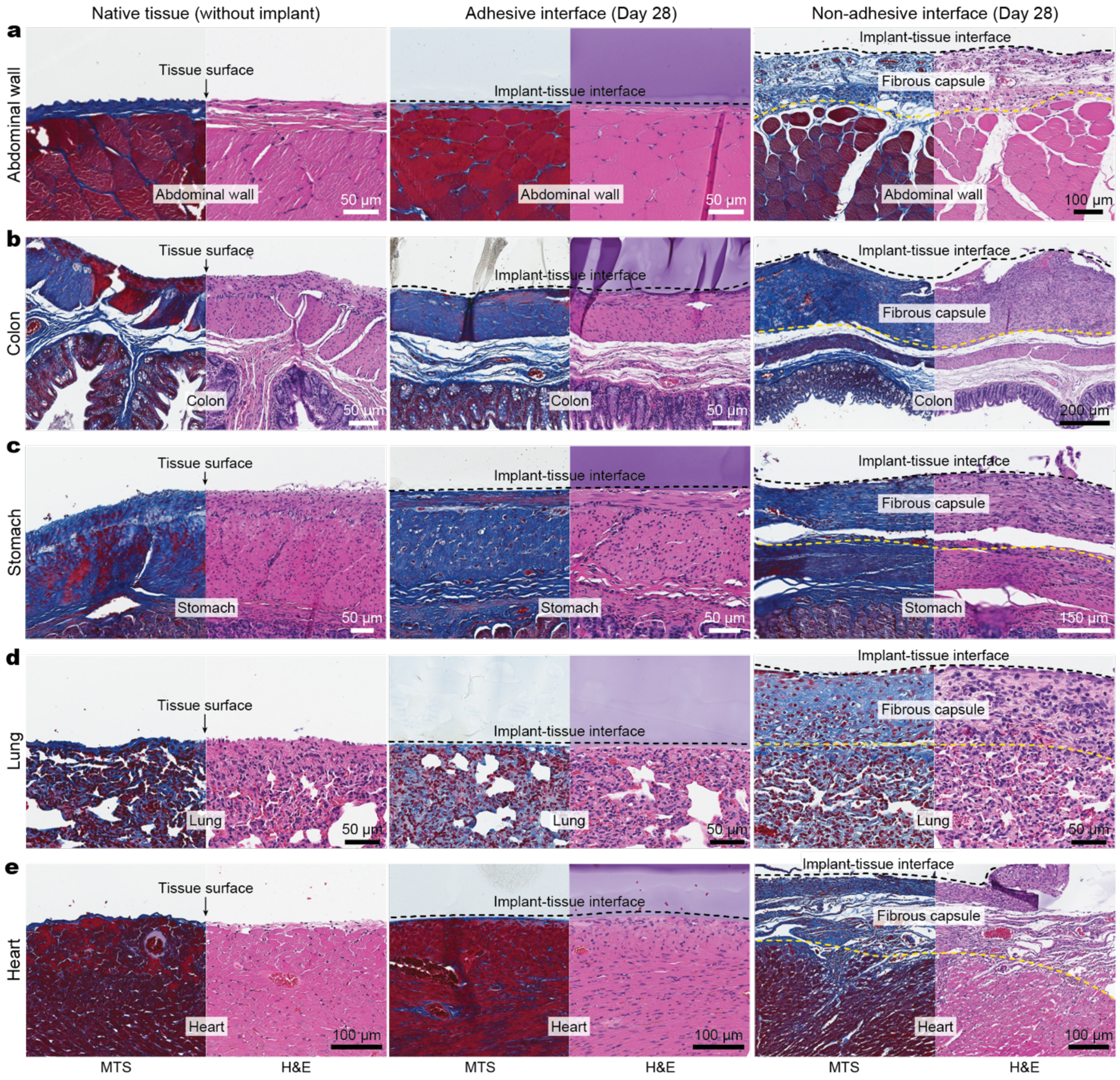

Supplementary Fig. 2 | Adhesive implant-tissue interface on diverse organs. a-e, Representative histology images stained with Masson's trichrome (MTS) and hematoxylin and eosin (H&E) for native tissue (left), adhesive implant (middle), and non-adhesive implant (right) collected on day 28 post-implantation to the abdominal wall (a), colon (b), stomach (c), lung (d), and heart (e). Black and yellow dotted lines in the images indicate the implant-tissue interface and the fibrous capsule-tissue interface, respectively. The experiment was repeated independently (n = 4 per group) with similar results.

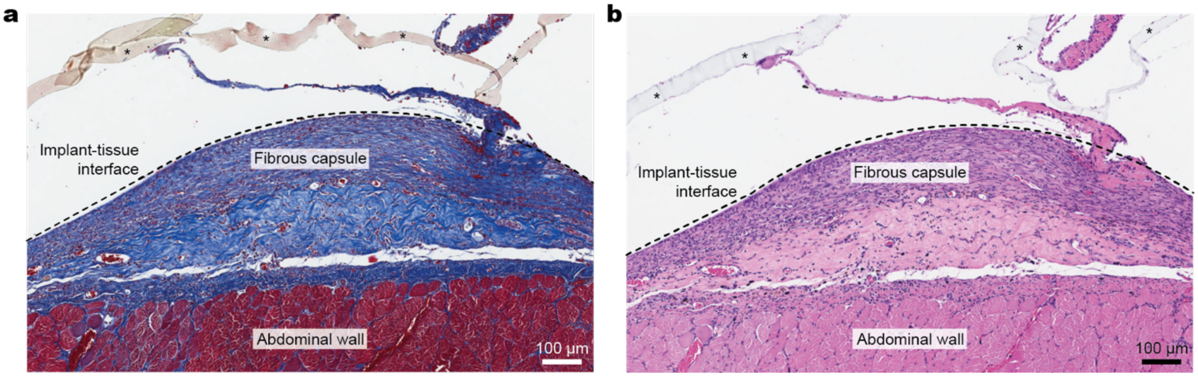

Supplementary Fig. 3 | Polyurethane mock device. a,**b**, Representative histology images stained with Masson's trichrome (a) and hematoxylin and eosin (b) of the polyurethane mock device collected on day 28 post-implantation to the abdominal wall. * in images indicates the implant; black dotted lines indicate the implant-tissue interface. The experiment was repeated independently (n = 4) with similar results.

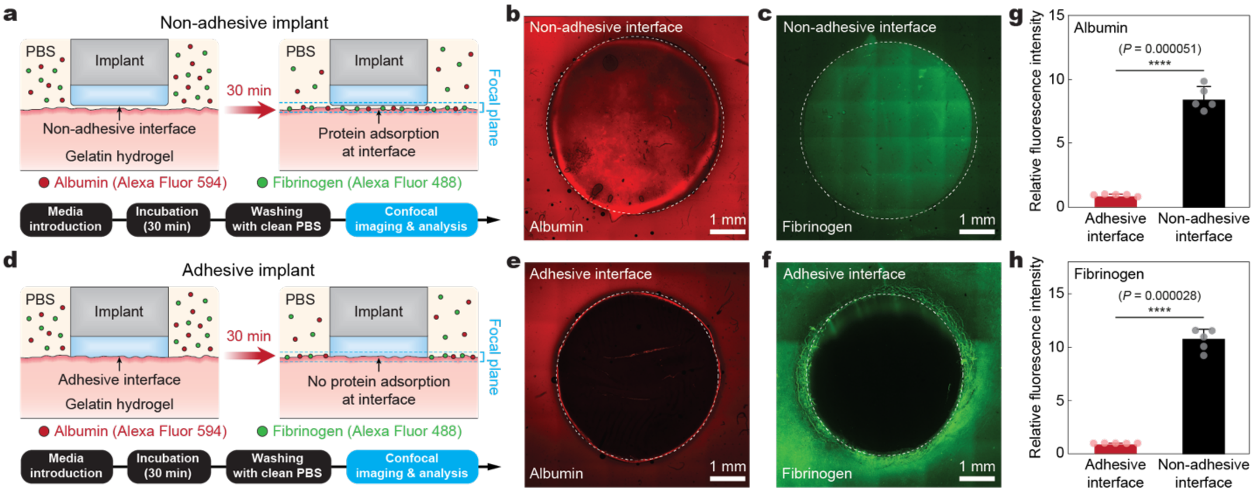

Supplementary Fig. 4 | In vitro protein adsorption assay. a-c, Schematic illustration of the experimental setup (a) and representative confocal microscope images for in vitro albumin (b) and fibrinogen (c) adsorption assay for the non-adhesive implant. **d-f**, Schematic illustration of the experimental setup (d) and representative confocal microscope images for in vitro albumin (e) and fibrinogen (f) adsorption assay for the adhesive implant. **g**,**h**, Relative fluorescence intensity at the implant-substrate interface measured 30 min after co-culture for albumin (g) and fibrinogen (h). Values in **g**,**h** represent the mean and the standard deviation ($n = 5$; independent samples). Statistical significance and P values are determined by two-sided unpaired t-tests; $^{****}P < 0.0001$.

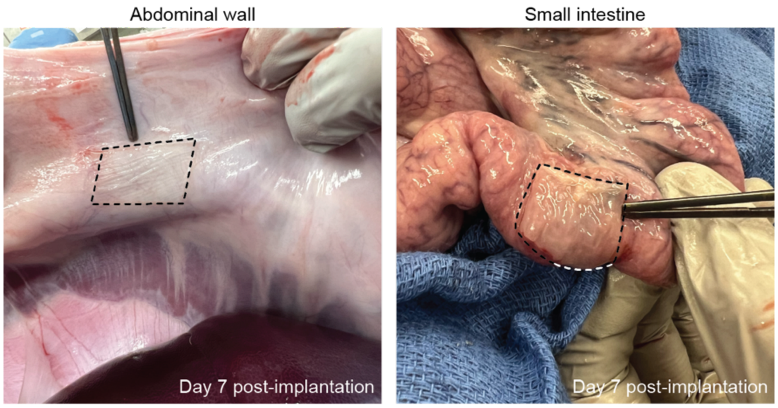

Supplementary Fig. 5 | In vivo implantation of the adhesive implants on porcine organs. Photographs of the porcine abdominal wall and small intestine on day 7 post-implantation for the adhesive implant. Black dotted lines in photographs indicate the boundary of implants.

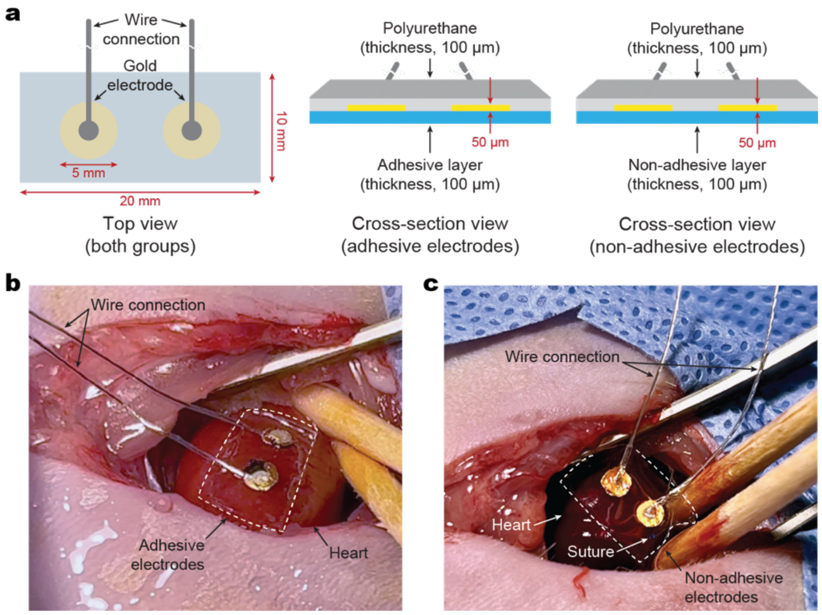

Supplementary Fig. 6 | Electrodes with the adhesive and non-adhesive interfaces. a, Schematic illustrations for electrodes with the adhesive and the non-adhesive interfaces. **b**,**c**, Photographs of electrodes with the adhesive interface (b) and the non-adhesive interface (c) implanted to the rat epicardial surface. White dotted lines in photographs indicate the boundary of implants.

Supplementary Table 1 l Luminex multiplex assays for protein profiling of rat abdominal wall tissues. Data were normalized per total amount of proteins extracted and are represented as the mean ± standard deviation (n = 3 tissue lysates; independent biological replicates). Statistical significance and P values are determined by two-sided unpaired t-tests; *$P < 0.05$ (vs. non-adhesive interface for the same time point).

Molecule (pg mg^{-1} protein)	Non-adhesive interface		Adhesive interface	
	Day 3	Day 7	Day 3	Day 7
IL-1α	6.87±2.61	10.47±5.38	3.87±0.45	8.75±5.08
IL-1β	107.65±129.51	125.97±130.24	20.95±4	26.77±8.04
IL-6	31.48±45.88	50.44±60	15.31±11.07	18.74±6.33
IL-12P70	1.05±0.23	1.18±0.32	1.74±0.28*	1.7±0.35
IL-17	3.18±0.34	4.04±1.2	3.22±0.47	3.74±0.2
IL-18	394.21±20.14	430.18±111.11	192.43±28.83*	145.5±12.14*
TNF-α	0.58±0.02	0.98±0.4	0.69±0.07	0.65±0.11
MCP-1	53.77±44.43	64.64±28.67	47.47±16.12	34.17±2.23
G-CSF	0.2±0.05	0.25±0.03	0.36±0.06*	0.34±0.06
IL-4	12.62±5.8	12.61±1.71	17.41±3.46	13.13±1.61
IL-10	4.64±2.63	9.93±11.36	3.03±0.35	2.31±0.25
CCL3	17.34±3.56	77.24±73.93	9.21±1.15*	20.74±14.28
CCL5	10.15±1.49	69.24±31.03	5.88±0.84*	38.57±25.39

Supplementary Table 2 | Mechanical properties of the implants in this study

Implant	Young's modulus	Thickness
Polyurethane	2.1 MPa	100 μm
Gold electrode	79 GPa	50 μm
