# Supplementary material for: Adhesive anti-fibrotic interfaces on diverse organs
Source: Nature. 2024 May 22;630(8016):360–7. doi: 10.1038/s41586-024-07426-9 (PMC11168934; doi:10.1038/s41586-024-07426-9)
Supplement: Supplementary file 2 — Reporting Summary [file 41586_2024_7426_MOESM2_ESM.pdf]

nature research

Corresponding author(s): Hyunwoo Yuk
Xuanhe Zhao

Last updated by author(s): Apr 8, 2024

# Reporting Summary

Nature Research wishes to improve the reproducibility of the work that we publish. This form provides structure for consistency and transparency in reporting. For further information on Nature Research policies, see Authors & Referees and the Editorial Policy Checklist.

## Statistics

For all statistical analyses, confirm that the following items are present in the figure legend, table legend, main text, or Methods section.

| n/a | Confirmed | |
|---|---|---|
| ☐ | ☒ | The exact sample size (*n*) for each experimental group/condition, given as a discrete number and unit of measurement |
| ☐ | ☒ | A statement on whether measurements were taken from distinct samples or whether the same sample was measured repeatedly |
| ☐ | ☒ | The statistical test(s) used AND whether they are one- or two-sided<br>*Only common tests should be described solely by name; describe more complex techniques in the Methods section.* |
| ☒ | ☐ | A description of all covariates tested |
| ☐ | ☒ | A description of any assumptions or corrections, such as tests of normality and adjustment for multiple comparisons |
| ☐ | ☒ | A full description of the statistical parameters including central tendency (e.g. means) or other basic estimates (e.g. regression coefficient) AND variation (e.g. standard deviation) or associated estimates of uncertainty (e.g. confidence intervals) |
| ☐ | ☒ | For null hypothesis testing, the test statistic (e.g. *F*, *t*, *r*) with confidence intervals, effect sizes, degrees of freedom and *P* value noted<br>*Give P values as exact values whenever suitable.* |
| ☒ | ☐ | For Bayesian analysis, information on the choice of priors and Markov chain Monte Carlo settings |
| ☒ | ☐ | For hierarchical and complex designs, identification of the appropriate level for tests and full reporting of outcomes |
| ☒ | ☐ | Estimates of effect sizes (e.g. Cohen's *d*, Pearson's *r*), indicating how they were calculated |

*Our web collection on statistics for biologists contains articles on many of the points above.*

## Software and code

Policy information about availability of computer code

| | |
|---|---|
| Data collection | No software used for data collection. |
| Data analysis | Microscopic images were analyzed by using ImageJ (Version: 2.1.0). All statistical analyses were performed by using GraphPad Prism (Version: 9.2.0). Raw sequencing data (.bcl files) generated from Illumina HiSeq were converted into fastq files and de-multiplexed using Illumina's bcl2fastq software (version 2.17). |

For manuscripts utilizing custom algorithms or software that are central to the research but not yet described in published literature, software must be made available to editors/reviewers. We strongly encourage code deposition in a community repository (e.g. GitHub). See the Nature Research guidelines for submitting code & software for further information.

## Data

Policy information about availability of data

All manuscripts must include a data availability statement. This statement should provide the following information, where applicable:
- Accession codes, unique identifiers, or web links for publicly available datasets
- A list of figures that have associated raw data
- A description of any restrictions on data availability

All data supporting the findings of this study are available within the Article and its Supplementary Information. The RNA-seq data generated in the present study were deposited in the Gene Expression Omnibus (GEO) with accession number 'GSE198219'. Additional raw data generated in this study are available from the corresponding authors upon reasonable request.

# Field-specific reporting

Please select the one below that is the best fit for your research. If you are not sure, read the appropriate sections before making your selection.

☒ Life sciences ☐ Behavioural & social sciences ☐ Ecological, evolutionary & environmental sciences

For a reference copy of the document with all sections, see nature.com/documents/nr-reporting-summary-flat.pdf

# Life sciences study design

All studies must disclose on these points even when the disclosure is negative.

| | |
|---|---|
| Sample size | The sample size for rodent and porcine studies were determined based on the literature with similar studies (Wu et al., Science Translational Medicine 14, eabh2857 (2022)).<br><br>For all rats in vivo studies, the appropriate sample size (n = 4 per each time point & group) were conducted to investigate foreign body reactions with implant on various organs used.<br><br>In vivo experiments on HuCD34-NCG mice (n = 5) and C57BL/6 mice (n = 6 per each time point) were conducted to investigate foreign body reactions with adhesive or non adhesive implant.<br><br>In vivo experiments on pig (n = 4) were conducted to investigate foreign body reactions with implant. |
| Data exclusions | No animal was excluded. |
| Replication | In vivo studies were reliably reproduced based on comparable histological assessment for each case by the blinded pathologist. All in vivo studies were independently performed with at least 1 day between surgeries. All attempts at replication were successful. |
| Randomization | All the tests were performed with randomly allocated experimental groups. |
| Blinding | All histological assessments were conducted by the blinded pathologist based on randomly mixed histological slides without informing type or study group of samples. All other measurements were conducted in a blinded fashion. |

# Reporting for specific materials, systems and methods

We require information from authors about some types of materials, experimental systems and methods used in many studies. Here, indicate whether each material, system or method listed is relevant to your study. If you are not sure if a list item applies to your research, read the appropriate section before selecting a response.

## Materials & experimental systems

| n/a | Involved in the study | |
|---|---|---|
| ☐ | ☒ | Antibodies |
| ☒ | ☐ | Eukaryotic cell lines |
| ☒ | ☐ | Palaeontology |
| ☐ | ☒ | Animals and other organisms |
| ☒ | ☐ | Human research participants |
| ☒ | ☐ | Clinical data |

## Methods

| n/a | Involved in the study | |
|---|---|---|
| ☒ | ☐ | ChIP-seq |
| ☒ | ☐ | Flow cytometry |
| ☒ | ☐ | MRI-based neuroimaging |

## Antibodies

| | |
|---|---|
| Antibodies used | Primary antibodies: Mouse anti-αSMA (ab7817, Abcam); Mouse anti-CD68 (ab201340, Abcam); Rabbit anti-CD3 (ab5690, Abcam); Rabbit anti-CD206 (ab64693, Abcam); Mouse anti-vimentin (ab8978, Abcam); Rabbit anti-iNOS (ab283655, Abcam); Mouse anti-iNOS (GTX60599, GeneTex); Rabbit anti-neutrophil elastase (bs-6982R, Bioss).<br><br>Secondary antibodies: Alexa Fluor 488 labeled anti-rabbit or anti-mouse secondary antibody (315-545-003, Jackson Immunoresearch), or Alexa Fluor 594 labeled donkey anti-mouse secondary antibody (715-586-151, Jackson Immunoresearch). |
| Validation | All antibodies are commercially available and have been tested by the manufacturer. Vendors and catalog numbers are listed above and validation can be found there.<br><br>Mouse anti-aSMA (ab7817, Abcam): This monoclonal antibody recognizes aSMA. Manufacturer-validated to react with Mouse, Rat, Rabbit, Human, Pig aSMA (https://www.abcam.com/alpha-smooth-muscle-actin-antibody-1a4-ab7817.html).<br><br>Mouse anti-CD68 (ab201340, Abcam): This monoclonal antibody recognizes CD68. Manufacturer-validated to react with Mouse, Rat, Human CD68 (https://www.abcam.com/cd68-antibody-c68684-ab201340.html). |

Rabbit anti-CD3 (ab5690, Abcam): This polyclonal antibody recognizes to CD3. Manufacturer-validated to react with Mouse, Rat, Human CD3 (https://www.abcam.com/cd3-antibody-ab5690.html).

Rabbit anti-iNOS (ab283655, Abcam): This multiclonal antibody recognizes to iNOS. Manufacturer-validated to react with Mouse, Rat, Human iNOS (https://www.abcam.com/inos-antibody-rm1017-ab283655.html)

Rabbit anti-CD206 (ab64693, Abcam): This polyclonal antibody recognized to CD206. Manufacturer-validated to react with Mouse, Rat, Human CD206 (https://www.abcam.com/mannose-receptor-antibody-ab64693.html).

Mouse anti-Vimentin (ab8978, Abcam): This monoclonal antibody recognized to Vimentin. Manufacturer-validated to react with Mouse, Rat, Human Vimentin (https://www.abcam.com/vimentin-antibody-rv202-cytoskeleton-marker-ab8978.html).

Mouse anti-iNOS (GTX60599, GeneTex): This monoclonal antibody recognized to iNOS. Manufacturer-validated to react with Mouse, Rat, Human iNOS (https://www.genetex.com/Product/Detail/iNOS-antibody-4E5/GTX60599?utm_source=listng&utm_medium=click&utm_id=AntibodyResource)

Rabbit anti-Neutrophil Elastase (bs-6982R-A488, BiossAntibodies): This polyclonal antibody recognized to neutrophil elastase. Manufacturer-validated to react with Human, Mouse, Rat neutrophil elastase (https://www.biossusa.com/products/bs-6982r-a488)

## Animals and other organisms

Policy information about studies involving animals; ARRIVE guidelines recommended for reporting animal research

| | |
|---|---|
| Laboratory animals | Female and male Sprague Dawley rats (12 weeks, 225-275g weight), were purchased from Charles River Laboratories.<br>Female and male C57BL/6 mice (6-8 weeks, 18-25g) were purchased from Jackson Laboratory.<br>Female HuCD34-NCG mice (16-18 weeks, 18-25g) were purchased from Charles River Laboratories.<br>Female Domestic pigs (20 weeks, 50 kg) were purchased from Manthei Hog Farm, LLC. |
| Wild animals | This study does not involve wild animals. |
| Field-collected samples | This study does not involve field-collected samples. |
| Ethics oversight | Animal procedures for rat and mice were reviewed and approved by the Massachusetts Institute of Technology Committee on Animal Care.<br><br>Animal procedures for pig were were reviewed and approved by the Mayo Clinic IACUC at Rochester. |

Note that full information on the approval of the study protocol must also be provided in the manuscript.
